# Maternal serum concentrations of vitamin D in pregnancy and preterm birth: a case-control study in Southern Sweden

**DOI:** 10.1007/s00394-025-03716-8

**Published:** 2025-05-31

**Authors:** Henrik Olstrup, Lars Rylander, Christian Lindh, Gunilla Malm, Andreas Vilhelmsson

**Affiliations:** Division of Occupational and Environmental Medicine, Department of Laboratory Medicine, Lund University, Lund, 223 63 Sweden

**Keywords:** Vitamin D, Logistic regression, Extreme preterm, Severe preterm, Late preterm, Scania

## Abstract

**Purpose:**

The purpose of this case-control study was to investigate the associations between maternal serum concentrations of vitamin D early in pregnancy and the occurrence of preterm birth.

**Methods:**

The study included 269 women (cases) whose children were born preterm (< 37 gestational weeks [gw]) and 332 women (controls) whose children were born term (≥ 37 gw). Among the cases, 59 were extreme preterm (< 28 gw), 74 severe preterm (28‒32 gw), and 136 late preterm (33‒36 gw). All women gave birth in Scania, the most Southern County of Sweden. Concentrations of 25-hydroxyvitamin D_3_ (vitamin D) in maternal serum collected early in pregnancy were analyzed using liquid chromatography-tandem-mass-spectrometry (LC/MS/MS). The serum concentrations of vitamin D were trichotomized based on the distributions among the controls (≤ 44.9; 45.0‒68.8; and ≥ 68.9 nmol/L) as well as dichotomized at a predefined cut-off (< 50 and ≥ 50 nmol/L). Logistic regression was used to estimate the associations between vitamin D and preterm births, partly when all cases were included in the analyses, and partly when the three different case groups were included separately. The category with the highest vitamin D concentration was used as a reference in the regression analyzes. The analyses were performed without as well as with adjustments for potential confounders.

**Results:**

When the category with the lowest vitamin D concentrations were compared with the reference category in the analyses where the vitamin D concentrations were trichotomized, no statistically significant associations were observed. However, among the extreme preterm an adjusted odds ratio of 1.93 (95% confidence interval 0.83‒4.48) was observed. The patterns were similar when 50 nmol/L was used as the cut-off.

**Conclusion:**

Although all comparisons gave adjusted odds ratios in the direction that low maternal vitamin D concentrations early in pregnancy increase the risk of preterm birth, none of these were statistically significant. Accordingly, the present study gives no to very weak support for an association.

## Introduction

Each year, an estimated 15 million babies are born preterm, and micronutrient deficiencies, including vitamin D deficiency, are often associated with adverse pregnancy outcomes [1]. Based on a cross-sectional study in Sweden, vitamin D deficiency (plasma concentrations of vitamin D < 50 nmol/l) was found to occur in 18% and 40% of the adult population during the summer and winter months, respectively [2]. In Sweden, preterm birth (births before 37 completed weeks of pregnancy) occurs in just over 5% of all births [3].

The role of vitamin D during pregnancy has been analyzed in several previous observational studies, with results that suggest a positive association between vitamin D deficiency and preterm birth [4]. In a systematic review and meta-analysis, the dose-response relationships between vitamin D status and the risk of low birth weight, macrosomia, preterm birth, small for gestational age, and intrauterine growth restriction were analyzed. An overall conclusion was that a sufficient level of vitamin D during pregnancy was protective against the risk of preterm birth, low birth weight, and small for gestational age [5]. However, regarding preterm birth, the authors stated that there was considerable heterogeneity between studies, and indications of positive publication bias.

The associations between vitamin D concentrations during pregnancy and preterm births have been investigated in a large number of studies. In a systematic review and meta-analysis, a statistically significant increased risk of preterm birth was observed among those women with a maternal serum concentration of vitamin D of less than 50 nmol/L taken before or at delivery [6]. An increased risk of preterm birth associated with vitamin D deficiency, and a decreased risk of preterm birth associated with vitamin D supplementation during pregnancy, were shown in a meta-analysis based on both observational studies and randomized control trials [7]. Vitamin D supplementation and its role in different pregnancy outcomes (preeclampsia, gestational diabetes mellitus, small for gestational age, low birth weight, preterm birth, birth weight, birth length, and cesarean section) was analyzed in a systematic review and meta-analysis based on randomized controlled trials. Supplementation with vitamin D was associated with higher circulating concentrations of vitamin D, greater birth weight and length, but it was not associated with other maternal and neonatal outcomes [8].

**Fig. 1:**
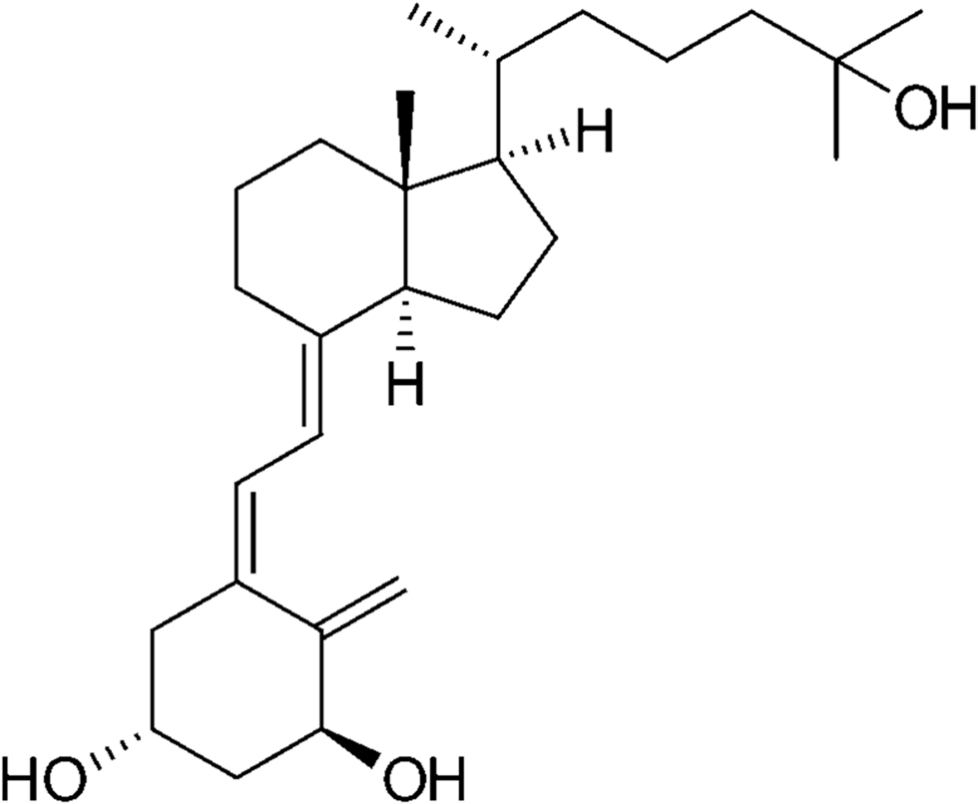
The chemical structure of calcitriol (1α,25-dihydroxyvitamin D)

Vitamin D constitutes a group of fat-soluble seco-steroids (steroids with a broken ring) with a lot of biological functions. Several forms of vitamin D exist, but D_3_ (cholecalciferol) constitutes the most active metabolite [9]. A particularly important role of vitamin D is to regulate the absorption and transportation of minerals (especially calcium, phosphorous, and magnesium) within the body, which are important in order to regulate bone metabolism and bone homeostasis [10, 11]. Vitamin D from chemical production in the skin or from diet is biologically inactive. It is activated by hydroxylation, which takes place in a two-step process in the liver and in the kidneys. In a first step, vitamin D is, under the influence of an enzyme, metabolized in the liver where 25-dihydroxyvitamin D is produced. In a second step, 25-dihydroxyvitamin D is, under the influence of an enzyme, metabolized in the kidneys where the hormonal form called 1α,25-dihydroxyvitamin D (calcitriol) (Fig. 1) is produced [12, 13].

Apart from its role in bone metabolism, vitamin D has other important roles. Hundreds of genes with the vitamin D receptor regulate, directly or indirectly, both cell cycling, cell proliferation, cell differentiation, cell death, innate and adaptive immunity, and brain development [14–16].

In terms of having a favorable fetal development, vitamin D has several biochemical roles. Apart from regulating the uptake of calcium by the fetus, vitamin D is also involved in fetal growth, development of the nervous system, lung maturation, and the function of the immune system [17]. Vitamin D regulates targets in the human placenta which involve a variety of both stimulatory and inhibitory actions. These actions work by vitamin D affecting genes, enzymes, hormones, and cytokines. These actions thereby affect biological networks including cell life and death, immune function, and bone and mineral metabolism [18].

Since vitamin D plays a crucial role regarding favorable fetal development, it is important to conduct research to prevent negative effects during pregnancy associated with inadequate levels of vitamin D.

Preterm birth is associated with a variety of negative health effects that can occur during lifetime. In terms of neurological and psychiatric outcomes, increased prevalence of disabilities including cerebral palsy, mental retardation, autism spectrum, and disorders of psychological development have been shown to occur among those born prematurely [19]. Chronic cardiovascular and kidney diseases are recognized as having early origins through altered developmental programming. They are in these cases caused by unfavorable environmental conditions during development, and preterm birth constitutes an adverse risk factor [20]. In addition, the respiratory system can be affected by preterm birth, and neonatal deaths among premature infants are usually caused by pulmonary diseases. Premature birth also means an increased risk of developing more chronic lung diseases [21]. Finally, the immune system can be affected by preterm birth. The immune system in premature infants has a smaller pool of certain types of white blood cells, which means a poorer defense against pathogens [22]. Consequently, when considering preterm births, their potential consequences later in life mean that it is important to take steps to avoid that from happening.

The purpose of this study was to analyze the associations between maternal serum concentrations of vitamin D (25-hydroxyvitamin D_3_) early in pregnancy and preterm birth among women in Scania, the most southern county in Sweden.

## Materials and methods

### Study group

This is a register and biobank-based case-control study. The participants consist of controls from an ongoing case-control study in Scania (the most southern county in Sweden) investigating the associations between environmental pollutants and cerebral palsy (CP) [23]. In that study, 368 singleton controls born between 1995 and 2014 were randomly selected from the Swedish Medical Birth Register. Among those, 13 were born preterm (before gestational week 37). In addition, since preterm birth is frequent among children diagnosed with CP [24], 277 additional singleton controls born preterm were randomly selected. This generated 292 cases (preterm birth) and 355 controls (term birth).

In the present study, 269 out of the 292 cases (92%) had enough serum samples in the biobank. The corresponding ratio among the controls were 332 out of 355 (94%). The reason for including the large number of extra preterm controls in the previous study regarding CP was the fact that a significant proportion of the children diagnosed with CP were born preterm. It should be noted that the gestational age distribution of preterm births should correspond to that among the CP cases. Hence, the frequency of sub-categories among the preterm cases in the present study (extreme [gestational week < 28, *n* = 59], severe [gestational week 28‒32, *n* = 74], and moderate to late preterm [gestational week 33‒36, *n* = 136]) does not match the general distribution among preterm births.

All women who participated in this study were informed that a part of their serum sample should be stored in a biobank for use in future research. Each participant gave a verbal informed consent at the time the blood sample was collected.

### Analyzes of vitamin D

The serum samples were collected from the Southern Sweden Maternity Cohort, which is a biobank that has stored serum samples collected from pregnant women since 1989. The samples were taken at approximately week 10‒14, when screening for viral infections and rubella [25]. This biobank covers more than 90% of all pregnancies in Scania.

Quantitative analyzes of vitamin D (25-hydroxyvitamin D_3_) were performed using methods previously described in Gustafsson et al. (2015) [26]. In short, internal standard were added to 0.1 mL aliquots of serum. The proteins were precipitated with acetonitrile, vigorously shaken for 30 min, and later centrifuged and analyzed using a LC/MS/MS (QTRAP 5500, AB Sciex, Framingham, MA, USA). The samples were prepared and analyzed in random order. Chemical blanks and quality control (QC) samples were included in all analytical batches. QC samples from Chromsystems Instruments & Chemicals (GmbH Gräfelfing, Germany) were within specified range and the coefficient of variation (CV) was between 8 and 13%.

### Other pregnancy information

Additional data early in pregnancy were collected from the Swedish Medical Birth Register (SMBR) including maternal body mass index (BMI, kg/m^2^), parity, maternal smoking (three categories: non-smokers, 1‒9 cig/day, and > 9 cig/day), and gender.

### Statistical analysis

Initially, comparisons of serum concentrations of vitamin D (measured as a continuous variable) between cases and controls were tested using Mann-Whitney tests. In addition to treat the cases as one group, the cases were divided into subgroups: (i) extreme preterm, (ii) severe preterm, and (iii) late preterm, and analyzes were performed for these case groups separately. In a second step, logistic regression was applied which generated odds ratios (ORs) and 95% confidence intervals (CIs). In these analyses, the concentrations of vitamin D were trichotomized based on the distribution among the controls (≤ 44.9; 45.0‒68.8; and ≥ 68.9 nmol/L) as well as dichotomized at a predefined cut-off (< 50 and ≥ 50 nmol/L) which was based on concentrations suggested in the literature [27]. The highest exposure category was used as the reference category. The crude analyses were completed with a priori defined model adjusting for maternal age (three categories: <31, 31‒34, and > 34 years), BMI early in pregnancy (three categories: <25, 25‒<30, ≥ 30 kg/m^2^), smoking early in pregnancy (two categories: yes and no), and parity (two categories: 1 and ≥ 2). However, it is important to stress that in some of the adjusted analyzes the cases were relatively few, and these results must be interpreted with caution. Statistical significance was defined as p-values below 0.05 or confidence intervals (CIs) not including 1.00. All analyzes were performed using SPSS software version 29.0 (IBM, Armonk, NY, USA).

## Results

The most pronounced differences regarding background characteristics between the cases and the controls were the higher fractions of mothers with overweight/obesity among the extreme and severe preterm case groups (Table 1). In addition, all case groups consisted of a greater proportion of first-time (primiparous) mothers in comparison with the control group.

**Table 1:** Background characteristics among 269 preterm cases and 332 controls. In addition, the cases are divided into three groups (extreme preterm, severe preterm, and late preterm)

		Mean/Median (Standard deviation, Interquartile range)			
	Extreme preterm < 28 weeks (*n* = 59)	Severe preterm 28‒32 weeks (*n* = 74)	Late preterm 33‒36 weeks (*n* = 136)	Total preterm (*n* = 269)	Controls (*n* = 332)
Maternal age at childbirth	30/30 (5, 7)	31/30 (5, 8)	30/30 (6, 8)	30/30 (5, 7)	32/32 (5, 6)
Calendar-year of childbirth	2006/2008 (5, 8)	2006/2007 (6, 9)	2006/2006 (5, 7)	2006/2007 (5, 7)	2004/2004 (5, 8)
Gestational days at partus	180/179 (9, 12)	211/212 (8, 15)	243/246 (11, 19)	221/224 (27, 46)	280/280 (9, 13)
		**n (%)**			
**BMI early in pregnancy (kg/m** ^**2**^ **)**					
< 25 25‒<30 ≥ 30 Missing	28 (53.8) 12 (23.1) 12(23.1) 7	39 (56.5) 20 (29.0) 10 (14.5) 5	81 (65.3) 28 (22.6) 15 (12.1) 12	148 (60.4) 60 (24.5) 37 (15.1) 24	207 (67.2) 73 (23.7) 28 (9.1) 24
**Parity**					
1 ≥ 2 Missing	34 (61.8) 21 (38.2) 4	41 (56.9) 31 (43.1) 2	75 (55.1) 61 (44.9) 0	150 (57.0) 113 (43.0) 6	144 (43.8) 185 (56.2) 3
**Smoking early in pregnancy**					
Non-smokers 1‒9 cig/day > 9 cig/day Missing	52 (94.5) 3 (5.5) 0 (0.0) 4	64 (90.1) 4 (5.6) 3 (4.2) 3	120 (90.2) 6 (4.5) 7 (5.3) 3	236 (91.1) 13 (5.0) 10 (3.9) 10	299 (92.0) 15 (4.6) 11 (3.4) 7
**Gender (boys)**	33 (55.9)	45 (60.8)	50 (51.5)	148 (55.0)	160 (48.2)

When vitamin D was analyzed as a continuous variable (Table 2), the concentrations did not significantly differ between the cases and the controls. Regardless of whether all cases were included, or only one of the subgroups was included, there were no statistically significant differences between the case groups and the controls (all p-values > 0.25 in Mann-Whitney tests).

**Table 2:** Maternal vitamin D serum concentrations (25-hydroxyvitamin D_3_, nmol/L) early in pregnancy among 269 preterm cases and 332 controls. In addition, the cases are divided into three groups based on severity

Group	*N*	Mean	Median	Min.	Max.	Standard deviation	25th percentile	75th percentile
**Cases**								
Extreme Preterm (weeks < 28) Severe Preterm (weeks 28‒32) Late Preterm (weeks 33‒36) Total **Controls** (weeks ≥ 37) **Cases + Controls**	59 74 136 269 332 601	54.6 57.7 55.8 56.1 57.4 56.8	49.8 55.1 54.8 54.4 56.1 54.9	11.7 5.1 5.8 5.1 4.5 4.5	161.2 220.6 140.1 220.6 151.6 220.6	26.6 31.3 28.3 28.7 26.4 27.4	35.9 37.6 35.8 37.3 39.3 37.9	67.6 72.7 74.2 70.8 75.0 74.5

When the lowest exposure category was compared with the highest exposure category, all adjusted analyzes generated odds ratios above 1.0 (Tables 3 and 4). However, none of these associations were statistically significant.

**Table 3:** The associations between maternal vitamin D concentrations (25-hydroxyvitamin D_3_, nmol/L) early in pregnancy and preterm birth. The cut-offs are trichotomized based on the distributions among the controls as well as on a predefined concentration (50 nmol/L). Odds ratios (OR) and 95% confidence intervals (CI) were obtained from single (unadjusted) and multiple (adjusted) logistic regressions

Vitamin D (nmol/L)	Cases (*n*)	Controls (*n*)	Unadjusted OR 95% CI	Adjusted ^a, b^ OR 95% CI
**Trichotomized**				
≤ 44.9 45.0‒68.8 ≥ 68.9	89 103 77	109 110 113	1.20 0.80‒1.79 1.37 0.93‒2.04 1.00 Ref	1.29 0.83‒2.02 1.40 0.91‒2.16 1.00 Ref
**Dichotomized**				
< 50 ≥ 50	118 151	136 196	1.13 0.81‒1.56 1.00 Ref	1.20 0.84‒1.72 1.00 Ref

^a^ Adjusted for maternal age (three categories: <31, 31‒34, and > 34 years), BMI early in pregnancy (three categories: <25, 25‒ <30, and ≥ 30 kg/m^2^ ), smoking early in pregnancy (two categories: yes and no), and parity (two categories: 1 and ≥ 2)

^b^ Only individuals with complete data were included

**Table 4:** The associations between maternal vitamin D concentrations (25-hydroxyvitamin D_3_, nmol/L) early in pregnancy and different preterm categories. The cut-offs are trichotomized based on the distributions among the controls as well as on a predefined concentration (50 nmol/L). Odds ratios (OR) and 95% confidence intervals (CI) were obtained from single (unadjusted) and multiple (adjusted) logistic regressions

Vitamin D (nmol/L)	Cases (*n*)			Controls (*n*)	Extreme Preterm (< 28 weeks)				Severe Preterm (28–32 weeks)				Late Preterm (33–36 weeks)			
					Unadjusted		Adjusted ^a, b^		Unadjusted		Adjusted ^a, b^		Unadjusted		Adjusted ^a, b^	
	< 28	28–32	33–36		OR	95% CI	OR	95% CI	OR	95% CI	OR	95% CI	OR	95% CI	OR	95% CI
≤ 44.9	20	21	48	109	1.60	0.76–3.36	1.93	0.83–4.48	1.04	0.54-2.00	1.26	0.61–2.61	1.16	0.71–1.89	1.15	0.67–1.96
45.0-68.8	26	32	45	110	2.06	1.00-4.20	1.96	0.86–4.47	1.56	0.85–2.88	1.80	0.91–3.55	1.08	0.66–1.76	1.11	0.65–1.88
≥ 68.9	13	21	43	113	1.00	Ref	1.00	Ref	1.00	ref	1.00	ref	1.00	ref	1.00	Ref
< 50	30	29	59	136	1.49	0.86–2.60	1.72	0.92–3.22	0.93	0.55–1.56	1.07	0.61–1.87	1.10	0.74–1.65	1.13	0.73–1.76
≥ 50	29	45	77	196	1.00	Ref	1.00	Ref	1.00	Ref	1.00	Ref	1.00	Ref	1.00	Ref

^a^ Adjusted for maternal age (three categories: <31, 31‒34, and > 34 years), BMI early in pregnancy (three categories: <25, 25‒ <30, and ≥ 30 kg/m^2^ ), smoking early in pregnancy (two categories: yes and no), and parity (two categories: 1 and ≥ 2)

^b^ Only individuals with complete data were included

## Discussion

### The calculated odds ratios and possible explanations

In this study, we wanted to investigate if vitamin D early in pregnancy was associated with preterm birth. When comparing the categories with the lower vitamin D concentrations with the category with the highest concentrations (reference category), all adjusted odds ratios pointed in the direction that low concentrations increased the risk of preterm birth. However, none of these odds ratios were statistically significant, giving no or very weak support for an association.

It is important to note here that the serum concentrations of vitamin D in this study were taken early in pregnancy, in the late first trimester and in the early second (week 10‒14). This is of importance, since concentrations of vitamin D measured at different time points during pregnancy, and their importance for pregnancy outcomes, have been analyzed in a few studies. For instance, a previous systematic review and meta-analysis covering different study designs (cohort, case-control, and cross-sectional studies) showed that vitamin D deficiency in middle pregnancy (second trimester) was likely to increase the risk of preterm birth, while no statistically significant associations were found regarding the first and third trimester [27]. In a post-hoc analysis, based on two randomized controlled vitamin D supplementation studies, the associations between serum concentrations of vitamin D at three time points (< 16 weeks, 16‒26 weeks, and ≥ 27 weeks) and preterm births were analyzed. The serum concentrations of vitamin D closest to delivery were more significantly associated with preterm births [28]. Consequently, the lack of statistically significant associations in the present study could be explained by the fact that vitamin D concentrations at the beginning of pregnancy do not have as great impact as during the latter part. However, a statistically significant association between the concentrations of vitamin D during the first trimester (8‒14 weeks of gestation) and preterm births was found among a group belonging to an ethnic minority in Canada [29]. A statistically significant association between serum concentrations of vitamin D, measured at or before 20 weeks of gestation, and an increased risk of preterm birth was also found in a study performed in U.S [30].

In our sample, the strongest associations, although not statistically significant, were found among the category “extreme preterm” (< 28 weeks). The point estimates for the odds ratios in the vitamin D concentrations categories of ≤ 44.9 and 45.0‒68.8 nmol/L had values in the range of 1.60 to 2.06 when vitamin D concentrations in the category ≥ 68.9 nmol/L was used as a reference. Likewise, the point estimates for the odds ratios in the vitamin D concentration category of < 50 nmol/L were 1.49 (unadjusted) and 1.72 (adjusted) when vitamin D concentrations in the category of ≥ 50 nmol/L was used as a reference. Pooled estimates from meta-analysis studies have shown an increased risk of preterm birth associated with serum concentrations of vitamin D < 50 nmol/L when compared to concentrations > 50 nmol/L [5, 6]. Regarding vitamin D and an optimal health from a more general perspective, the greatest risks for cancer, infections, cardiovascular and metabolic diseases have also been shown in association with serum concentrations of vitamin D below 50 nmol/L [31].

The aforementioned studies indicate that there may be a critical lower limit at serum concentration of vitamin D at approximately 50 nmol/L, both in terms of preterm birth and a general state of health and disease prevention. When considering an upper limit, there is probably an approximate value where the health-promoting effects begin to decline. The associations between age-adjusted hazard ratios for all-cause mortality and serum concentrations of vitamin D were analyzed in a meta-analysis based on 32 studies. Pooled estimates showed that serum concentrations of vitamin D less than 75 nmol/L were associated with greater mortality in comparison with concentrations greater than 75 nmol/L. However, the dose-response curve based on all studies combined showed that the estimated hazard ratio was no longer significantly different from 1.0 at a serum concentration of vitamin D of 90 nmol/L [32]. However, an optimal serum concentration of vitamin D for maximum health has been difficult to determine [33, 34]. When considering the results presented in this study, there are indications that the risk of preterm births are higher below the cut-off values of both 50 nmol/L and 68.9 nmol/L, and that applies in particular to the category “extreme preterm”. However, the relatively small number of participants makes the associations somewhat uncertain.

### The results in this study in comparison with the results in similar studies

The results in this study with a lack of statistically significant associations, but pointing in the direction of an increased risk of preterm birth in association with lower serum concentrations of vitamin D measured early in pregnancy, can be compared with the results from studies with similar study designs. When considering studies that have analyzed vitamin D concentrations during pregnancy and its relation to an increased risk of preterm births, the results point in different directions. Statistically significant associations between relatively lower serum concentrations of vitamin D and an increased risk of preterm birth have been shown in a majority of the studies that have analyzed the relationships [1, 35–43]. Positive but not statistically significant associations have, similar to this present study, been found in two studies [39, 44]. The lack of clear statistical associations has also been shown in several studies [35, 39, 45-50], and finally, a significantly negative association was found in one study by Zhou et al. (2014) [51].

When considering the associations between vitamin D concentrations and preterm births according to the studies described above, a majority of these indicate that there is an association. Possible biological mechanisms behind this association include upregulated genes associated with both vitamin D insufficiency and preterm birth which give rise to dysregulation of specific immune response pathways [52]. However, several studies including the present one have not found any significant associations. Positive but not statistically significant associations can possibly be explained by a relatively small data base. Statistically significant negative associations can be explained by confounding by other factors, as shown in the study by Zhou et al. (2014) [51] where higher prevalence of preterm birth among women with relatively higher concentrations of vitamin D can probably be explained by older age and higher body mass index within this group [51].

### Strengths and limitations of this study

There are both strengths and limitations of our study that need to be considered. One important strength is that our data were based on a population-based biobank with 90% coverage in Scania in southern Sweden, which means that the risk of selection bias was small. The tests were taken at approximately the same time, and they were analyzed in a lab with high-quality measuring equipment´s. Based on previous studies from our research group, it has been shown that even if serum samples of vitamin D were stored for a long time, they match the expected seasonal pattern, indicating a high reliability [53, 54]. A similar seasonal pattern was shown for the serum samples of vitamin D in this present study, although these data have not been presented. Another strength was the possibility to adjust for a number of possible confounders, and also that preterm births could be divided into different categories and analyzed accordingly.

A weakness of this study is that we only have one sample of serum concentrations of vitamin D from each participant taken early in pregnancy that has not been stored in a systematic way. For that reason, we had no possibility to study the importance of vitamin D at different time periods during pregnancy. Another weakness is that we lacked the statistical power to detect relatively small differences (Appendix Tables A1 and A2).

Moreover, this study was based on a relatively small number of participants which gave relatively uncertain statistical relationships. Other potential confounding factors that have not been included could also be of importance; for instance, data regarding heritability of preterm birth, medical and obstetrical history, diet during pregnancy, ethnicity, and socioeconomic status. Lower vitamin D concentrations among lower socioeconomic groups have been shown among women of childbearing age in rural northern China [55]. Differences in the effects of vitamin D concentrations on preterm births among different ethnic groups have also been shown to occur based on a multicenter prospective cohort of pregnant women [39].

## Conclusions

This case-control study showed positive but not statistically significant associations between the concentrations of vitamin D early in pregnancy and preterm birth. An overall conclusion is that there is only a very weak support for a relationship. The odds of having a preterm birth were most clear and robust in the category with the most serious cases of preterm birth (extreme preterm < 28 weeks). Adjustments for a number of covariates did not to any great extent change the odds ratios of having a preterm birth. In this study, the serum concentrations of vitamin D were only measured early in pregnancy, and it could potentially have influenced the results if the concentrations during the latter part of pregnancy would have a greater impact on the risk of preterm births.

## Appendix A

**Table A1:** The associations between maternal vitamin D concentrations (25-hydroxyvitamin D_3_, nmol/L) in early pregnancy and preterm birth. Similar to Table 3, the cut-offs were trichotomized based on the distributions among the controls as well as on a predefined concentration (50 nmol/L). In addition, the vitamin D concentrations were divided into quartiles based on the controls divided into four equal groups, and divided into quartiles with a cut-off of 50 nmol/L between quartile 2 and 3. Odds ratios (OR) and 95% confidence intervals (CI) were obtained from single (unadjusted) and multiple (adjusted) logistic regressions

Vitamin D (nmol/L)	Cases (*n*)	Controls (*n*)	Unadjusted OR 95% CI	Adjusted^a, b^ OR 95% CI
≤ 44.9	89	109	1.20 0.80–1.79	1.29 0.83–2.02
45.0-68.8	103	110	1.37 0.93–2.04	1.40 0.91–2.16
≥ 68.9	77	113	1.00 Ref	1.00 Ref
≤ 39.1	79	83	1.36 0.86–2.15	1.64 0.99–2.73
39.2–56.1	66	83	1.14 0.71–1.83	1.26 0.75–2.10
56.2–75.0	66	83	1.14 0.71–1.81	1.25 0.75–2.10
≥ 75.1	58	83	1.00 Ref	1.00 Ref
< 25	37	36	1.47 0.83–2.60	1.69 0.89–3.22
26–49	81	100	1.16 0.74–1.81	1.34 0.82–2.18
50–74	93	113	1.18 0.76–1.82	1.32 0.82–2.13
≥ 75	58	83	1.00 Ref	1.00 Ref
< 50	118	136	1.13 0.81–1.56	1.20 0.84–1.72
≥ 50	151	196	1.00 Ref	1.00 Ref

^a^ Adjusted for maternal age (three categories: <31, 31‒34, and > 34 years), BMI early in pregnancy (three categories: <25, 25‒ <30, and ≥ 30 kg/m^2^ ), smoking early in pregnancy (two categories: yes and no), and parity (two categories: 1 and ≥ 2).

^b^ Only individuals with complete data were included

**Table A2:** The associations between maternal vitamin D concentrations (25-hydroxyvitamin D_3_, nmol/L) early in pregnancy and different preterm categories. Similar to Table 4, the cut-offs were trichotomized based on the distributions among the controls as well as on a predefined concentration (50 nmol/L). In addition, the vitamin D concentrations were divided into quartiles based on the controls divided into four equal groups, and divided into quartiles with a cut-off of 50 nmol/L between quartile 2 and 3. Odds ratios (OR) and 95% confidence intervals (CI) were obtained from single (unadjusted) and multiple (adjusted) logistic regressions

Vitamin D (nmol/L)	Cases (*n*)			Controls (*n*)	Extreme Preterm (< 28 weeks)				Severe Preterm (28–32 weeks)				Late Preterm (33–36 weeks)			
					Unadjusted		Adjusted^a, b^		Unadjusted		Adjusted^a, b^		Unadjusted		Adjusted^a, b^	
	< 28	28–32	33–36		OR	95% CI	OR	95% CI	OR	95% CI	OR	95% CI	OR	95% CI	OR	95% CI
≤ 44.9	20	21	48	109	1.60	0.76–3.36	1.93	0.83–4.48	1.04	0.54-2.00	1.26	0.61–2.61	1.16	0.71–1.89	1.15	0.67–1.96
45.0-68.8	26	32	45	110	2.06	1.00-4.20	1.96	0.86–4.47	1.56	0.85–2.88	1.80	0.91–3.55	1.08	0.66–1.76	1.11	0.65–1.88
≥ 68.9	13	21	43	113	1.00	ref	1.00	ref	1.00	ref	1.00	ref	1.00	ref	1.00	Ref
≤ 39.1	17	20	42	83	1.89	0.80–4.80	3.02	1.09–8.40	1.18	0.58–2.40	1.52	0.69–3.35	1.31	0.76–2.28	1.46	0.79–2.69
39.2–56.1	18	19	29	83	2.00	0.85–4.71	2.06	0.72–5.87	1.12	0.54–2.30	1.36	0.62–2.98	0.91	0.50–1.63	1.04	0.55–1.96
56.2–75.0	15	18	33	83	1.67	0.69–4.02	2.48	0.87–7.04	1.06	0.51–2.20	1.07	0.47–2.43	1.03	0.58–1.83	1.10	0.59–2.07
≥ 75.1	9	17	32	83	1.00	Ref	1.00	ref	1.00	Ref	1.00	Ref	1.00	Ref	1.00	Ref
< 25	5	8	24	36	1.28	0.40–4.09	1.64	0.41–6.51	1.08	0.43–2.74	1.31	0.47–3.66	1.73	0.90–3.34	1.91	0.91–4.02
26–49	25	21	35	100	2.31	1.02–5.21	3.21	1.21–8.56	1.02	0.51–2.07	1.26	0.59–2.73	0.91	0.52–1.59	1.01	0.55–1.86
50–74	20	28	45	113	1.63	0.71–3.77	2.13	0.78–5.86	1.21	0.62–2.36	1.35	0.64–2.85	1.03	0.60–1.76	1.15	0.64–2.06
≥ 75	9	17	32	83	1.00	ref	1.00	ref	1.00	ref	1.00	ref	1.00	ref	1.00	ref
< 50	30	29	59	136	1.49	0.86–2.60	1.72	0.92–3.22	0.93	0.55–1.56	1.07	0.61–1.87	1.10	0.74–1.65	1.13	0.73–1.76
≥ 50	29	45	77	196	1.00	Ref	1.00	Ref	1.00	Ref	1.00	Ref	1.00	Ref	1.00	Ref

^a^ Adjusted for maternal age (three categories: <31, 31‒34, and > 34 years), BMI early in pregnancy (three categories: <25, 25‒ <30, and ≥ 30 kg/m^2^ ), smoking early in pregnancy (two categories: yes and no), and parity (two categories: 1 and ≥ 2)

^b^ Only individuals with complete data were included
